# Gender Identity Profiles in Autistic and Non‐Autistic Cisgender and Gender Diverse Youth, and Their Caregivers

**DOI:** 10.1002/aur.70142

**Published:** 2025-11-14

**Authors:** Matthew C. Fysh, Aimilia Kallitsounaki, David M. Williams, Eilis Kennedy, Lauren Spinner

**Affiliations:** ^1^ Tavistock and Portman NHS Foundation Trust London UK; ^2^ Autism Research Centre, Department of Psychiatry University of Cambridge Cambridge UK; ^3^ School of Psychology University of Kent Canterbury UK; ^4^ Department of Clinical Educational and Health Psychology University College London London UK

**Keywords:** autism disorder, caregivers, family, gender dysphoria, gender identity, phenotype

## Abstract

This preregistered study examined whether the gender identity phenotype differs between autistic and non‐autistic children and adolescents, as well as whether gender identity traits aggregate similarly within their families. Study 1 involved four matched groups of autistic and non‐autistic gender diverse youth referred to a UK specialist gender clinic, as well as cisgender autistic and non‐autistic youth (*n* = 45 per group). Participants completed measures of gender typicality, discontentedness, anticipated future identity, and (parent‐reported) dysphoria. Despite large and significant differences between cisgender and gender diverse youth across all gender‐related measures, there were no significant differences between autistic and non‐autistic participants within either gender group. Study 2 assessed recalled childhood gender behaviors and current gender dysphoria in the caregivers of participants from each group (*N* = 203). Caregivers of gender‐referred youth, regardless of autism status, reported higher current dysphoric traits than caregivers of cisgender youth, but no differences were observed in recalled childhood gender‐related behavior. Overall, the findings indicate that the gender phenotype of autistic youth is comparable to that of non‐autistic youth within the same gender identity group, challenging the assumption that gender diversity in autism arises from different underlying mechanisms. Clinically, these results support equitable access to gender‐related care for autistic and non‐autistic gender diverse youth.

**Summary**

- This study found that autistic and non‐autistic gender diverse children show similar patterns of gender identity that contrast with the experience of gender identity that autistic and non‐autistic cisgender children report.
- Subtle differences in self‐reported experience of gender identity were also observed in the caregivers of gender diverse children compared to the caregivers of cisgender children, regardless of whether their child was autistic.
- These findings suggest that gender diversity in autism is not fundamentally different and support equal access to gender‐related care for all youth.

## 1 Introduction

Autism spectrum disorder (ASD) is a neurodevelopmental condition involving social‐communication difficulties and restricted, repetitive behaviors and interests (APA 2022). Emerging research suggests gender diversity (a mismatch between sex assigned at birth and gender identity; Butler 2020) tends to be more prevalent among autistic individuals than non‐autistic peers (Janssen et al. 2016; May et al. 2017; McPhate et al. 2021; Strang et al. 2014; Hisle‐Gorman et al. 2019; Pecora et al. 2020; Walsh et al. 2018). Gender diversity may be accompanied by clinically significant distress (i.e., gender dysphoria; APA 2013), sometimes leading to referral to specialist gender services.

Only a few studies have examined the behavioral manifestations of gender identity and diversity in community (i.e., non‐gender clinic‐referred) samples of autistic people. In *adults*, George and Stokes (2018) found higher gender dysphoria in autistic than non‐autistic individuals, though dysphoria scores were likely inflated in the autistic group because one‐third had a non‐cisgender identity. Although, among exclusively cisgender groups, Cooper et al. (2018) and Kallitsounaki and Williams (2022) reported that autistic adults expressed a weaker gender identity than non‐autistic peers. Thus, even in the absence of gender diversity or dysphoria, autistic individuals may experience a less strongly defined sense of gender identity than non‐autistic people.

In *children/adolescents* (henceforth described as “youth” to distinguish children and adolescents from adults), Corbett et al. (2023) found that parents of autistic children reported their children to have significantly higher levels of body incongruence/dysmorphia than parents of non‐autistic children, particularly in children assigned female at birth. Also, autistic children self‐reported feeling less like their sex and more likely to feel nonbinary (i.e., neither male nor female) than non‐autistic children (see Corbett et al. 2024 for an analysis of the developmental trajectories shown by participants on these measures). In contrast, Mo et al. (2024) found no group differences in gender‐related behavior across children with autism, ADHD, or typical development using the Gender Identity Questionnaire (a parent report measure of children's toy, playmate, and activity preferences, expressed identities, and body incongruence/dysphoria).

Discrepancies across studies may reflect differences in sample composition. Most studies have not distinguished between children who explicitly identify as gender diverse and those who identify as cisgender and so it is possible that observed group differences are primarily driven by a higher proportion of gender diverse individuals in autism groups. To our knowledge, no study has directly compared gender phenotype in exclusively cisgender autistic and non‐autistic children—a gap the current study addresses.

Although it is generally considered that gender identity is more variable in autistic than non‐autistic people, the origins of this variability remain unclear. Some have suggested that features of autism, such as cognitive rigidity (De Vries et al. 2010; Pasterski et al. 2014; VanderLaan et al. 2015; Zucker et al. 2017), social‐communication difficulties (Strang, Janssen, et al. 2018; Strang, Powers, et al. 2018; Cooper, Mandy, et al. 2023), resistance to social conventions (Strang et al. 2023), and sensory sensitivities (Cooper, Butler, et al. 2023), may contribute to the emergence of gender diversity. Strang et al. (2023) noted that autism may predispose gender diversity through specific developmental mechanisms:

…that autism itself might influence the gender experience is important to consider for clinical practice […] that the autistic experience itself might color the experience and interpretation of gender suggests that this might be a specific pathway to gender diversity (p. 10)

This raises important clinical questions about how best to support autistic youth with gender‐related concerns. If gender diversity in this group stems from autism‐specific mechanisms, they may face barriers to care including the invalidation of their gender identity (Strang, Janssen, et al. 2018; Strang, Powers, et al. 2018). Indeed, some gender diverse autistic individuals report concealing their autism diagnosis to avoid discrimination (Cooper et al. 2022). A key approach to addressing these questions is to compare the gender profiles, or “phenotypes,” of autistic and non‐autistic *gender diverse* youth. Divergences between these groups could suggest distinct developmental pathways. Only a handful of studies have explored this.

In a community sample of *adults*, Cooper et al. (2018) found no differences between autistic and non‐autistic gender diverse participants in self‐reported strength of gender identity, gender self‐esteem (i.e., positivity about one's gender group), or masculinity/femininity. Similarly, Kallitsounaki and Williams (2022) observed similar levels of self‐reported gender dysphoria and recalled childhood gender‐typed interests and behaviors among autistic and non‐autistic gender diverse adults.

In *adolescents*, Tollit et al. (2024) compared the strength of gender identity, dysphoria, and gender expression between clinic‐referred participants scoring above (*n* = 239) and below (*n* = 283) the clinical cut‐off on the Social Responsiveness Scale‐2 (SRS‐2). No differences emerged in self‐reported gender identity, dysphoria, or parent‐reported gender diverse behaviors. However, adolescents with high autism traits reported greater body dysphoria and higher rates of social transition. These findings suggest broad similarities in gender phenotype, although subtle differences may reflect distinct developmental pathways.

Yet, while Tollit et al.'s study was impressive in size and scope, some caution is warranted when interpreting their findings due to their use of the SRS‐2 to assign participants into “high” and “low” autistic trait groups, rather than basing group assignment on clinical diagnoses. Although the SRS‐2 has reasonable psychometric properties (Wigham et al. 2012), scores are influenced by factors beyond autism (Hus et al. 2013) and do not reliably distinguish autism from other neurodevelopmental or psychiatric conditions (Cholemkery et al. 2014; Kerr‐Gaffney et al. 2021; South et al. 2017). Consequently, it is unclear whether participants exhibiting high autism trait scores in Tollit et al.'s study truly had autism or whether the group's elevated SRS‐2 scores instead reflected overlapping traits associated with other conditions. This concern is amplified by the finding that the high trait group also showed significantly higher rates of depression (73% vs. 26%), anxiety (65% vs. 17%), and ADHD (21% vs. 1%) than the low trait group. These complexities make it difficult to interpret gender‐related findings when groups differ on other clinical measures and have been defined using a screening tool rather than clinical diagnosis.

Taking a more rigorous diagnostic approach, but in a smaller study, Fischbach et al. (2025) compared gender diverse autistic (*n* = 27) and non‐autistic (*n* = 39) adolescents recruited from a mixture of community and clinic settings. They found no differences in the age of gender identity emergence, binary versus nonbinary identities, dysphoria, or dimensions of gender expression, although autistic participants were more likely to use gender‐neutral pronouns. These results suggest similarities in gender identity variation across groups.

Given the uncertainties surrounding Tollit et al.'s (2024) results, and the relatively modest sample size in Fischbach et al.'s (2025) study, it is unclear whether gender‐related behavior is similar in autistic and non‐autistic gender diverse children. In Study 1, we compared gender identity profiles in groups of gender diverse autistic and non‐autistic youth referred to a UK specialist gender clinic, as well as cisgender autistic and non‐autistic youth. Four measures captured behavioral aspects of gender typicality, contentedness, dysphoria, and identity. Using a four‐group crossed design, we investigated whether (a) autistic and non‐autistic gender diverse youth show similar gender phenotypes and (b) cisgender autistic youth show subtle differences in gender identity compared to cisgender non‐autistic peers. In Study 2, we examined current gender dysphoria and recalled childhood gender identity in caregivers (primarily mothers) to assess whether gender identity variation aggregates within families of autistic versus non‐autistic gender diverse children. If gender identity variation stems from an autism‐specific pathway in autistic people, unlike non‐autistic people, we would expect (i) differences between autistic and non‐autistic gender diverse children in the gender phenotype, (ii) cisgender autistic children to show increased gender identity variability than cisgender non‐autistic children, and (iii) different patterns of familial transmission of gender identity.

## 2 Study 1: Method

### 2.1 Participants

Participants were 259 youth aged 7–16 years, assigned to one of four groups: autistic gender‐referred, non‐autistic gender‐referred, autistic cisgender, and non‐autistic cisgender. Gender‐referred groups (*n* = 117) were recruited from a prospective longitudinal study of youth referred to a UK specialist gender clinic and social media. Cisgender groups (*n* = 142) were recruited from the community. Exclusions included one participant with an IQ below 70, 24 participants who did not complete key experimental measures, and 15 children who were recruited from the general population (i.e., were not referred to a gender clinic), but who reported a gender identity incongruent with their sex assigned at birth. This resulted in a final sample of 219 participants.

Cognitive ability was assessed using the Vocabulary and Matrix Reasoning subtests of the Wechsler Abbreviated Scale of Intelligence—Second Edition (WASI‐II; Wechsler 2011). Subtest scores were converted to *t* scores and combined into an FSIQ‐2 composite. A matching script in R (see Supporting Information for details) was then used to remove participants iteratively until the four groups had equal numbers of participants assigned male and female at birth, and were matched on age and FSIQ. To confirm this, a 2 (gender group) × 2 (diagnostic status) ANOVA was conducted. The analysis yielded nonsignificant main effects and interactions (*p*s ≥ 0.106, *ƞ*_*p*_^2^ ≤ 0.02; BF_10_ ≤ 0.67).^1^ Each matched group contained 45 participants (49% assigned male at birth; see Table 1).

**TABLE 1:** Participant characteristics in study 1.

Variable	Cisgender		Gender‐referred	
	Non‐ASD	ASD	Non‐ASD	ASD
	*M* (SD)	*M* (SD)	*M* (SD)	*M* (SD)
Age	12.51 (1.60)	12.09 (1.88)	12.38 (1.77)	12.89 (2.36)
FSIQ2	108.42 (12.05)	103.27 (14.96)	103.58 (11.05)	104.24 (13.49)
Verbal *t*	55.89 (7.24)	51.22 (9.58)	53.38 (8.11)	53.00 (8.31)
Nonverbal *t*	53.89 (8.87)	52.60 (10.27)	50.93 (8.07)	51.96 (8.95)
BOSA^a^	—	8.79 (2.83)	—	8.49 (2.79)
ADI‐R (domain A)^b^	—	17.40 (5.60)	—	16.74 (6.74)
ADI‐R (domain B)^b^	—	14.33 (4.20)	—	13.33 (4.99)
ADI‐R (domain C)^b^	—	6.22 (2.23)	—	5.88 (2.46)
ADI‐R (domain D)^b^	—	2.40 (1.25)	—	2.23 (1.73)

*Note:* FSIQ2 = full scale IQ‐2; verbal *t =* WASI‐II vocabulary subtest *t* score; non‐verbal *t =* WASI‐II matrix reasoning subtest *t* score; BOSA = Brief Observation of Symptoms of Autism; ADI‐R (domains A, B, C, and D) = Autism Diagnostic Interview‐Revised (social difficulties; communication difficulties, RRBIs, and developmental difficulties evident at or before 36 months, respectively).

^a^ Cisgender ASD: *n* = 43; gender‐referred ASD: *n* = 41.

^b^ Cisgender ASD: *n* = 45; gender‐referred ASD: *n* = 43.

All participants in the autism groups had a formal ASD diagnosis, except eight who were undergoing assessment. These eight were retained as results were unchanged when excluded. Specifically, no previously significant *p* values became nonsignificant, and no nonsignificant *p* values became significant, when they were excluded. Additionally, all effect sizes remained within the same interpretive categories (i.e., small/medium/large), indicating that their inclusion did not meaningfully influence the overall findings. Autism features were assessed using the Brief Observation of Symptoms of Autism (BOSA; Dow et al. 2022; Lord et al. 2020) in 88/90 autistic participants and the Autism Diagnostic Interview‐Revised (ADI‐R; Rutter et al. 2003) in 84/90 (participant unavailability meant that some did not complete the BOSA and ADI). The BOSA is a virtual observational measure derived from the Autism Diagnostic Observation Schedule Second Edition (ADOS‐2; Lord et al. 2012), while the ADI‐R is a structured caregiver interview. The ADI comprises four subscales, corresponding to social difficulties, communication difficulties, restricted and repetitive behaviors and interests (RRBIs), and developmental difficulties evident at or before 36 months, respectively. Ninety‐one percent of participants scored above the clinical cut‐off on the BOSA (≥ 6) or ADI‐R (domains A, B, C, and D; ≥ 10, ≥ 8, ≥ 3, ≥ 1, respectively). Additionally, autistic cisgender and autistic gender‐referred groups were matched on both measures (all *p*s ≥ 0.307, *d*s ≤ 0.22, BF_10_s ≤ 0.35). Measures such as the ADI and BOSA are intended to inform, but not replace, clinical judgment, and low scores do not preclude a formal diagnosis.

All participants in the gender clinic‐referred groups reported a gender identity that was incongruent with their sex assigned at birth, apart from four (three autistic and one non‐autistic), who reported a gender identity congruent with their sex assigned at birth. These four were retained in the sample because they were referred to the clinic on the basis of gender‐related concerns, showing a history of gender diversity, even if their current self‐identification aligned with their sex assigned at birth. More importantly, no results changed meaningfully when these four participants were excluded (i.e., no *p* values crossed significance thresholds, and no effect sizes changed categories).

### 2.2 Materials

#### 2.2.1 Anticipated Future Gender Identity

Participants were asked, “What do you expect to feel like on the inside when you grow up?” (Olson et al. 2015). Responses options were boy, girl, neither, both, it changes over time, or don't know. Responses were coded as congruent (gender matched sex assigned at birth), incongruent (opposite binary gender), or other (all other responses).

#### 2.2.2 Gender Discontentedness

Participants were reminded of their sex assigned at birth: “When a baby is born, the doctor says the baby is a boy or girl based on the genitals (private parts) they were born with. When you were born the doctor said you were a baby [boy/girl].” They were then asked (1) “How much do you like being a [boy/girl]?,” referring to their sex assigned at birth, and (2) “How much would you like to be a [girl/boy]?,” referring to their opposite binary gender. Responses were rated on a 5‐point scale (1 = not at all to 5 = extremely). The second question was reverse‐coded, such that higher average scores across questions indicated greater contentedness with sex assigned at birth.

#### 2.2.3 Self‐Perceived Gender Typicality

Ten statements assessed how typical participants felt their preferences and appearance were for their sex assigned at birth (e.g., “I like to dress the same way as most [girls/boys]”) (Patterson 2012). Responses were rated from “Really Not True” to “Really True” on a 4‐point scale. Higher mean scores across items indicated greater gender typicality.

#### 2.2.4 Gender Dysphoria

Completed by parents about their children, this measure assessed dysphoria based on DSM‐5 criteria (Kennedy et al. 2021). One version was completed by parents about children aged 7–11 years and comprised nine items (e.g., “my child rejects toys, games or activities typically linked to their birth‐assigned gender”). Another version was completed by parents about children aged 12+ years and comprised seven items focused on gender identity and desire to change physical sex characteristics (e.g., “my child expresses a desire to get rid of their primary and/or secondary sex characteristics or a desire to prevent the development of anticipated sexual characteristics”). Responses were rated from 1 (strongly disagree) to 5 (strongly agree). Higher scores reflected greater dysphoria.

All measures were completed remotely via Zoom or Microsoft Teams over two sessions. Screen‐sharing was not used during measure completion (except for WASI subtests), but the experimenter was present to answer questions.

### 2.3 Ethics

Ethical approval was obtained from (a) Kent Psychology Research Ethics Committee (reference number: 202216553002907588) and (b) HRA and London–Hampstead Research Ethics Committee (reference number: 22/LO/0805). All materials were informed by a patient and public involvement (PPI) advisory group, and researchers were trained in safeguarding and held up‐to‐date DBS clearance.

### 2.4 Statistical Analyzes

The study, including the analysis plan, was preregistered on OSF (https://osf.io/dznkv/?view_only=49159395f86541f5863c937cac1887f2). Deviations from the preregistration are described in the Supporting Information. Effect sizes were reported using partial eta squared (*ƞp* ^2^; small = 0.01, medium = 0.06, large = 0.14) or Cramér's *V* (small = 0.07, medium = 0.21, large = 0.35). Bayesian analyzes assessed the strength of evidence for alternative versus null hypotheses (Dienes 2014). BF_10_ > 1 provides support for the alternative (substantial > 3, strong > 10, very strong > 30) and BF_10_ < 1 provides support for the null (substantial < 0.33, strong < 0.10, very strong < 0.03). Bayesian tests were run in JASP (v0.19.3) and R (v4.3.3). Note that Bayes factors were not computed for simple main effects due to the absence of a reliable method that retains the pooled error terms from the full ANOVA model, which is necessary to ensure consistency with the frequentist simple main effects analysis reported in the manuscript. Finally, note that all ANOVA findings reported are based on parametric ANOVAs. ANOVA is robust to violations of normality and homogeneity of variance, particularly when sample sizes are equal as they are in our study (see Glass et al. 1972). Nonetheless, all outcomes flagged by diagnostics for violation of assumptions were verified using the *WRS2* package in R. This package is designed specifically for this purpose, enabling the computation of robust ANOVAs with trimmed means to guard against violations of homogeneity and departure from normality (see Mair and Wilcox 2020). These robust analyzes produced identical patterns of results as the parametric tests, confirming the stability of results reported below (see Supporting Information for details).

## 3 Study 1: Results

### 3.1 Anticipated Future Gender Identity

**FIGURE 1:**
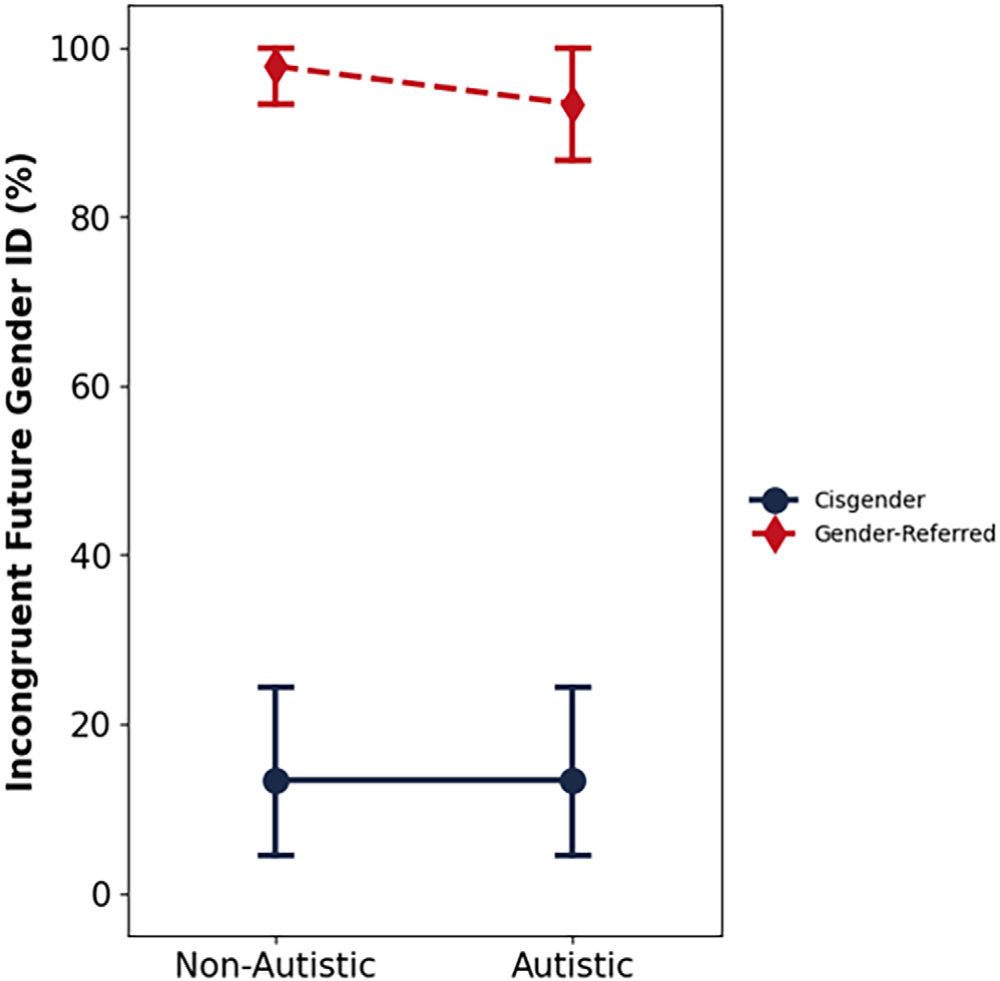
Cross‐subject mean percentage of participants in each group who anticipated an incongruent future gender identity. Error bars denote 95% confidence intervals.

Table 2 shows the distribution of participants' anticipated future gender identity across four groups: non‐autistic cisgender, autistic cisgender, non‐autistic gender‐referred, and autistic gender‐referred children. Due to the small number of cisgender participants reporting an incongruent future gender identity, “incongruent” and “other” responses were merged into a single category (see Figure 1) and were analyzed via two binomial logistic regression models. Model 1 contained the predictors gender group (cisgender vs. gender‐referred) and diagnostic status (autistic vs. non‐autistic), and Model 2 specified an interaction between these variables.

**TABLE 2:** Anticipated future gender identity across the four participant groups.

Variable	Cisgender			Gender‐referred		
	Non‐ASD	ASD	Total	Non‐ASD	ASD	Total
	*n*	*n*	*n*	*n*	*n*	*n*
Gender—future						
Congruent	39	39	78	1	3	4
Incongruent	0	1	1	33	31	64
Other	6	5	11	11	11	22

Both Models 1 and 2 accounted for a significant proportion of the variance in anticipated future gender identity, *χ* ^2^ = 144.98, *p* < 0.001 and *χ* ^*2*^ = 145.79, *p* < 0.001, respectively. However, the difference between Models 1 and 2 was trivial and nonsignificant, *χ* ^*2*^ = 0.81, *p* = 0.368, suggesting that Model 2's inclusion of an interaction term did not enhance the predictive capacity of the model. For this reason, Model 1 was retained in favor of Model 2, which explained between 55% (Cox & Snell) and 74% (Nagelkerke) of the variance in responses (pseudo‐*R* ^2^).

Gender group was a strong and significant predictor of reporting an *incongruent* future gender identity, OR = 142.06, *p* < 0.001, 95% CI: 43.69, 461.94, BF_10_ > 30, whereas diagnostic status did not significantly predict future gender identity, OR = 0.75, *p* = 0.597, 95% CI: 0.27, 2.15, BF_10_ = 0.34. Hence, gender‐referred children were far more likely than cisgender children to anticipate an incongruent future identity in both diagnostic groups (see Figure 1) Due to only one out of 45 autistic gender‐referred children (i.e., 2%) anticipating a congruent *future* identity, and likewise three out of 45 non‐autistic gender‐referred children (i.e., 7%) anticipating a future congruent identity, this analysis was repeated using Firth's penalized regression to account for near‐perfect separation. This did not alter the outcome of the analysis; group remained a significant predictor, OR = 180.28, *p* < 0.001, 95% CI = (37.83, 1808.38), diagnostic group remained a nonsignificant predictor, OR = 1.00, *p* > 0.99, 95% CI = (0.30, 3.30), and the interaction was ordinal (non‐crossing) and nonsignificant, OR = 0.41, *p* = 0.432, 95% CI = (0.03, 3.68).

### 3.2 Gender Discontentedness

**FIGURE 2:**
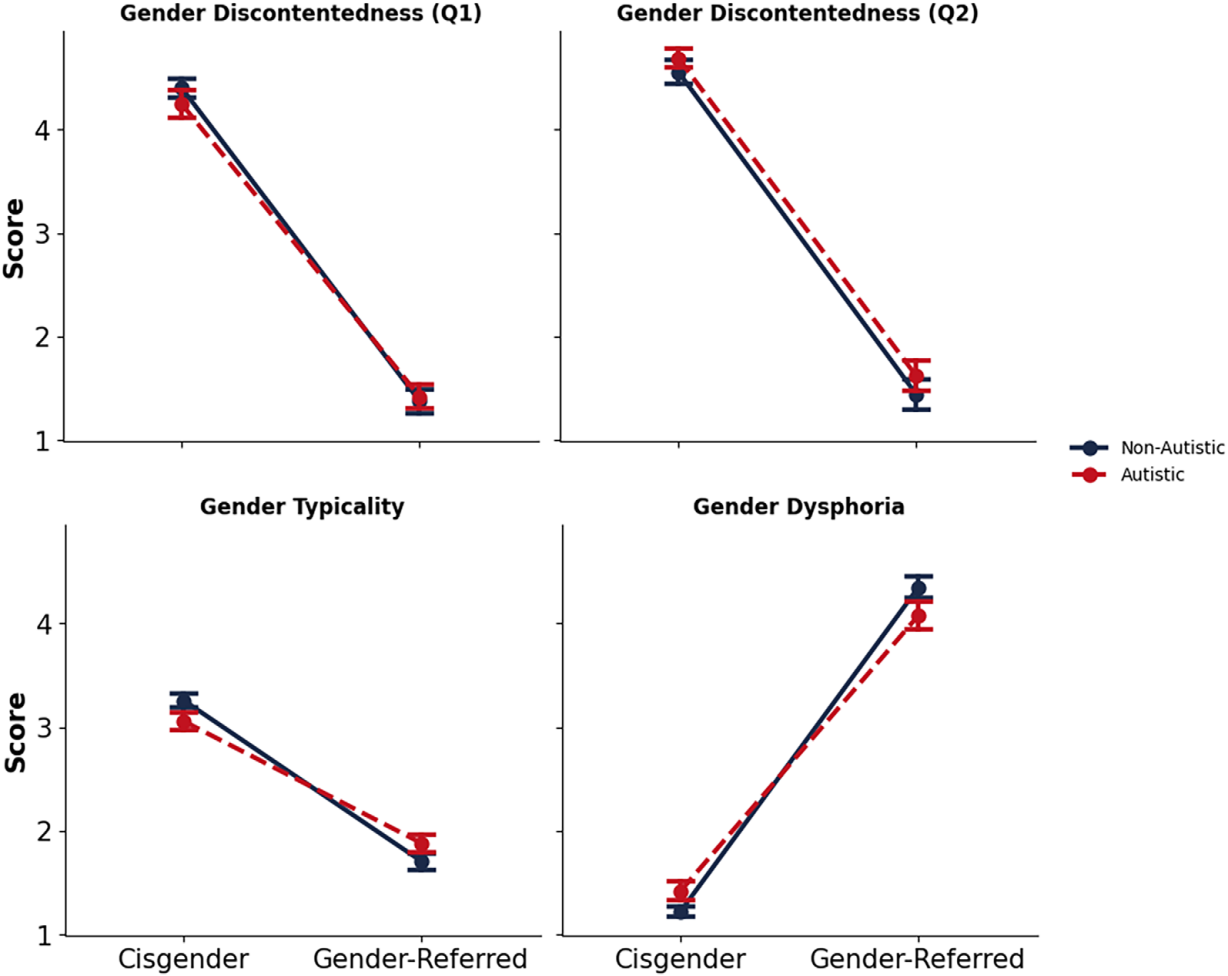
Cross‐subject means on the gender discontentedness items (top), and on the gender typicality and gender dysphoria scales (bottom left and bottom right, respectively). Autistic participants are denoted by the dashed line, and non‐autistic participants are denoted by the solid line. Error bars denote standard error of the mean.

Mean scores for questions 1 (contentedness with one's sex assigned at birth) and 2 (desire to be the opposite sex) are visualized in Figure 2 and were analyzed via separate 2 (gender group) × 2 (diagnostic status) ANOVAs. For question 1, a main effect of gender group reflected significantly higher contentedness in cisgender children (*M* = 4.32, SD = 0.76) than in gender‐referred children (*M* = 1.40, SD = 0.79), *F*(1, 176) = 634.05, *p* < 0.001, *ƞ*_*p*_^2^ = 0.78, BF_10_ > 30. There was no effect of diagnostic status, *F*(1, 176) = 0.23, *p* = 0.633, *ƞ*_*p*_^2^ < 0.01, BF_10_ = 0.17, or an interaction, *F*(1, 176) = 0.74, *p* = 0.390, *ƞ*_*p*_^2^ < 0.01, BF_10_ = 0.29.

For question 2, a main effect of gender group reflected a significantly stronger desire to be the opposite gender in gender‐referred children (*M* = 1.53, SD = 0.99) than in cisgender children (*M* = 4.62, SD = 0.68), *F*(1, 176) = 597.63, *p* < 0.001, *ƞ*_*p*_^2^ = 0.77, BF_10_ > 30. There was no effect of diagnostic status, *F*(1, 176) = 1.52, *p* = 0.220, *ƞ*_*p*_^2^ = 0.01, BF_10_ = 0.19, or an interaction, *F*(1, 176) = 0.03, *p* = 0.861, *ƞ*_*p*_^2^ < 0.01, BF_10_ = 0.22.

### 3.3 Gender Typicality

Mean gender typicality scores (see Figure 2) were analyzed via a 2 (gender group) × 2 (diagnostic status) ANOVA. The main effect of diagnostic status was nonsignificant, *F*(1, 176) = 0.02, *p* = 0.878, *ƞ*_*p*_^2^ < 0.01, BF_10_ = 0.16. However, a significant main effect of gender group emerged, *F*(1, 176) = 292.47, *p* < 0.001, *ƞ*_*p*_^2^ = 0.62, BF_10_ > 30, along with a significant interaction, *F*(1, 176) = 5.55, *p* = 0.020, *ƞ*_*p*_^2^ = 0.03, BF_10_ = 2.42.

Simple main effects analysis showed no significant difference in gender typicality between autistic and non‐autistic gender‐referred children, *F*(1, 176) = 2.43, *p* = 0.121, *ƞ*_*p*_^2^ = 0.01, or the cisgender children, *F*(1, 176) = 3.15, *p* = 0.078, *ƞ*_*p*_^2^ = 0.02. In contrast, cisgender children, both autistic and non‐autistic, reported significantly higher gender typicality than their gender‐referred peers (autistic: *F*(1, 176) = 108.72, *p* < 0.001, *ƞ*_*p*_^2^ = 0.38; non‐autistic: *F*(1, 179) = 189.29, *p* < 0.001, *ƞ*_*p*_^2^ = 0.52). The interaction appeared driven by a larger gender group effect among non‐autistic children (*ƞ*_*p*_^2^ = 0.52) than autistic children (*ƞ*_*p*_^2^ = 0.38), though this difference was modest and nonsignificant.

### 3.4 Gender Dysphoria

Parent‐reported gender dysphoria scores (see Figure 2) were analyzed via a 2 (diagnostic status) × 2 (gender group) ANOVA. The main effect of diagnostic group was nonsignificant, *F*(1, 176) = 0.14, *p* = 0.713, *ƞ*_*p*_^2^ < 0.01, BF_10_ = 0.16. However, the effect of gender group was significant, *F*(1, 176) = 812.05, *p* < 0.001, *ƞ*_*p*_^2^ = 0.82, BF_10_ > 30, as was the interaction, *F*(1, 176) = 5.31, *p* = 0.022, *ƞ*_*p*_^2^ = 0.03, BF_10_ = 2.20.

Simple main effects analysis showed that gender‐referred children, both non‐autistic and autistic, had significantly higher dysphoria than their cisgender peers (non‐autistic: *F*(1, 176) = 474.37, *p* < 0.001, *ƞ*_*p*_^2^ = 0.73; autistic: *F*(1, 176) = 342.99, *p* < 0.001, *ƞ*_*p*_^2^ = 0.66). In contrast, diagnostic status had no significant effect among cisgender, *F*(1, 176) = 1.88, *p* = 0.173, *ƞ*_*p*_^2^ = 0.01, or gender‐referred children, *F*(1, 176) = 3.57, *p* = 0.060, *ƞ*_*p*_^2^ = 0.02. Thus, autistic and non‐autistic participants showed equivalent levels of gender dysphoria within each gender group.^2^

## 4 Study 1: Discussion

This study observed consistent differences between cisgender and gender‐referred children across self‐report measures of future gender identity, gender contentedness and gender typicality, as well as parent‐reported dysphoria. Specifically, these differences were characterized by gender‐referred children expressing lower typicality and higher discontentment with their sex assigned at birth compared to cisgender children, as well as greater dysphoria and a rejection of their sex assigned at birth as their future gender identity. Crucially, however, there were no significant effects of diagnostic group on any measure. Hence, autistic gender‐referred participants resembled non‐autistic gender‐referred participants on all measures, just as autistic cisgender participants resembled non‐autistic cisgender participants. As such, we found no evidence that the gender phenotype of autistic participants differed from non‐autistic children of the same gender identity.

In Study 2, we investigated recalled childhood gender identity issues, as well as current feelings of gender dysphoria, in the caregivers of participants from Study 1. It is unclear whether gender identity issues aggregate in the families of gender diverse children, so the first aim of Study 2 was to investigate this. The second and main aim was to investigate the extent to which any pattern of aggregation differs in the families of autistic versus non‐autistic youth. If gender identity variation has a different basis in autistic people than in non‐autistic people, then different patterns of familial aggregation might be expected.

## 5 Study 2: Method

### 5.1 Participants

Caregivers of children and adolescents from four groups participated: autistic gender‐referred, non‐autistic gender‐referred, autistic cisgender, and non‐autistic cisgender (*N* = 253). Twelve participants who did not complete key experimental measures, as well as 15 participants from the cisgender groups whose child either reported a non‐cisgender identity or did not complete the gender identity measure, were excluded. After matching groups on caregiver age and sex assigned at birth using an R script (S1), the final sample included 51 caregivers of cisgender non‐autistic participants (*M*_age_ = 43.39, SD = 4.48), 51 caregivers of cisgender autistic participants (*M*_age_ = 43.18, SD = 6.34), 51 caregivers of gender‐referred non‐autistic participants (*M*_age_ = 43.92, SD = 6.27), and 50 caregivers of gender‐referred autistic participants (*M*_age_ = 44.96, SD = 4.64). Ninety‐eight percent of caregivers were assigned female at birth, and all reported a cisgender identity apart from two in the autism gender‐referred group.

### 5.2 Materials

#### 5.2.1 Recalled Childhood Gender Identity/Gender Role Questionnaire (RCGI)

The RCGI retrospectively measures gender‐incongruent feelings and play behaviors during childhood (0–12 years) (Zucker et al. 2006). We used 18 of the original 23 items, focusing on behaviors linked to gender roles and identity. Each item was rated on a 5‐point scale (1 = incongruent, 5 = congruent), with 12 items allowing a “not applicable” option. Male and female versions of the RCGI were administered to assigned male and assigned female caregivers, respectively. Scores reflected the mean of all completed items.

#### 5.2.2 Gender Identity/Gender Dysphoria Questionnaire for Adolescents and Adults (GIDYQ‐AA)

This 27‐item self‐report scale assesses the frequency of gender‐related thoughts, feelings, and behaviors (Deogracias et al. 2007). Items were rated from 1 (never) to 5 (always), with a lower mean score across items indicating greater gender dysphoria. Participants completed sex‐specific versions of the questionnaire.

## 6 Study 2: Results

### 6.1 Past Gender‐Typed Behavior

RCGI data (see Table 3) were analyzed via a 2 (child's diagnostic status: ASD/non‐ASD) × 2 (child's gender group: Cisgender/gender‐referred) ANOVA. The effect of child's diagnostic status was nonsignificant, *F*(1, 199) = 0.06, *p* = 0.807, *ƞ*_*p*_^2^ < 0.01, BF_10_ = 0.16, as was the effect of child's gender group, *F*(1, 199) = 1.36, *p* = 0.244, *ƞ*_*p*_^2^ = 0.01, BF_10_ = 0.29, and the interaction, *F*(1, 199) = 1.61, *p* = 0.207, *ƞ*_*p*_^2^ = 0.01, BF_10_ = 0.43.

**TABLE 3:** Means and standard deviations (in parentheses) for the RCGI and GIDYQ‐AA.

Measures	Cisgender		Gender‐referred	
	Non‐ASD	Autistic	Non‐ASD	Autistic
RCGI	3.56 (0.61)	3.65 (0.58)	3.57 (0.62)	3.43 (0.71)
GIDYQ‐AA	4.89 (0.17)	4.84 (0.17)	4.73 (0.47)	4.68 (0.50)

Abbreviations: GIDYQ‐AA, Gender Identity/Gender Dysphoria Questionnaire for Adolescents and Adults; RCGI, Recalled Childhood Gender Identity/Gender Role Questionnaire.

### 6.2 Current Gender Dysphoria

A 2 (child's diagnostic status) × 2 (child's gender group) ANOVA did not reveal an effect of child's diagnostic status, *F*(1, 199) = 0.91, *p* = 0.341, *ƞ*_*p*_^2^ = 0.01, BF_10_ = 0.23, or an interaction, *F*(1, 199) = 0.00, *p* = 0.991, *ƞ*_*p*_^2^ < 0.01, BF_10_ = 0.24. However, a significant effect of child's gender group was observed, *F*(1, 199) = 9.84, *p* = 0.002, *ƞ*_*p*_^2^ = 0.05, BF_10_ = 14.81, reflecting higher GIDYQ‐AA scores in parents of cisgender children than in parents of gender‐referred children (see Table 3).

## 7 Study 2: Discussion

There were no significant effects of the child's gender identity group (cisgender/gender‐referred) or diagnostic status (autistic/non‐autistic) on the caregiver's recalled memories of childhood feelings of gender incongruence. In contrast, current gender dysphoric traits were significantly more numerous in the caregivers of gender‐referred children than in the caregivers of cisgender children. Crucially, however, there was no effect of the child's diagnostic status on the caregiver's current gender dysphoric traits. Hence, current gender dysphoric traits appeared to aggregate in families of gender‐referred children, irrespective of whether the child was autistic or not.

## 8 General Discussion

The primary aim of these studies was to compare the gender phenotype of autistic and non‐autistic children, adolescents, and their caregivers. In Study 1, we examined whether (a) cisgender autistic children show subtle behavioral signs of gender variation despite their expressed cisgender identity, and (b) gender diverse autistic children display a similar profile of gender identity variation as non‐autistic gender diverse children.

Regarding (a), cisgender autistic and non‐autistic children showed broad similarities in gender identity presentation. Differences between groups on gender typicality, contentedness, dysphoria, and future identity were nonsignificant and associated with small effect sizes. This suggests that clinicians should not assume gender identity will necessarily be atypical in an autistic child. Interestingly, the finding contrasts with findings from studies of cisgender adults, which have found self‐reported gender identity to be significantly weaker in autistic than non‐autistic participants (Cooper et al. 2018; Kallitsounaki and Williams 2022). Of course, gender identity continues to evolve into adulthood and it may be that the subtle gender identity variation observed in cisgender autistic adults emerges later in development, only when gender identity becomes more socially defined and the pressure to conform to normative gender roles increases. In late adolescence and early adulthood, autistic adults might perceive their gender identity as less aligned with normative standards and therefore self‐report a weaker identification with their sex assigned at birth.

Regarding aim (b), Study 1 found that the gender phenotype of autistic gender diverse participants was practically indistinguishable from that of non‐autistic gender diverse participants. While this does not exclude the possibility of distinct underlying bases for gender diversity across autism and non‐autism groups, such consistent behavioral presentation across contentedness, future identity, typicality, and dysphoria would not be expected if different mechanisms were involved. Even less expected would be similar patterns of familial aggregation.

In Study 2, we examined recalled childhood gender identity issues and current gender dysphoria in caregivers. Cisgender caregivers of both autistic and non‐autistic gender diverse youth reported significantly greater dissatisfaction with their sex assigned at birth than caregivers of cisgender children. While GIDYQ scores in the caregivers of gender‐referred children did not indicate high levels of dysphoria in this group, they were elevated relative to levels self‐reported by caregivers of cisgender children. This pattern parallels findings in autism research, where non‐autistic relatives of autistic individuals often show elevated ASD traits (Bolton et al. 1994; Piven et al. 1997). It aligns with twin studies that report concordance between twins for gender identity variation (Coolidge et al. 2002; Diamond 2013; Karamanis et al. 2022) and moderate heritability of gender nonconformity (Burri et al. 2011), although it contrasts with findings from non‐twin siblings (Gülgöz et al. 2019). Greater similarity among twins than non‐twin siblings may account for differences between these studies. Why familial aggregation was evident in caregivers in the current study but not in siblings in other studies remains unclear.

At the same time, other studies have indicated that parents of gender diverse children themselves undergo a conceptual shift in their understanding of gender (Bull et al. 2022; Sweder et al. 2024). Given that parents of gender‐referred children in the current study did not recall gender‐incongruent desires or behaviors in their own childhood, yet diverged from parents of cisgender children when considering current feelings of incongruence, it is possible that elevated scores on the GIDYQ were an upstream consequence of their child's gender nonconformity.

Two points should be considered when evaluating these findings and their subsequent interpretations. First, the relatively modest sample size in Study 2 limits statistical power, although the detection of a group effect on caregiver GIDYQ is reassuring. Second, nearly all caregivers in Study 2 were assigned female at birth, limiting conclusions about caregivers assigned male at birth. Nonetheless, it is important that aggregation patterns were equivalent between caregivers of autistic and non‐autistic gender diverse youth.

Together, these findings build on those of Tollit et al. (2024) and Fischbach et al. (2025) in showing that the gender diversity phenotype is highly similar across autistic and non‐autistic youth. The current study was larger than Fischbach et al.'s and more diagnostically rigorous than Tollit et al.'s. Moreover, it is the only study to use a four‐group design, enabling a comprehensive understanding of how autism and gender identity independently and jointly relate to gender‐related behavior and feelings.

Overall, the findings provide evidence that gender diversity in autistic youth does not merely result from autism itself, but reflects variation in gender identity that is equivalent to that seen in non‐autistic youth. Of course, an analysis of behavior cannot alone provide definitive evidence about the developmental mechanisms or pathways that underlie that behavior, because the same behavior can stem from multiple distinct underlying pathways (e.g., Toth and Cicchetti 1996). For example, the use of self‐report measures leaves open the possibility that other factors, such as alexithymia, could mask true differences between autistic and non‐autistic people in gender identity phenotype. However, showing equivalence between autistic and non‐autistic gender diverse, as well as cisgender, children across *multiple* dimensions of gender‐related behavior reduces the likelihood that different mechanisms underpin gender identity in each group. Nonetheless, future studies might usefully replicate the findings of Study 1 using indirect measures of gender identity (e.g., the Implicit Association Test) that are less susceptible to biases related to self‐presentation and introspection (e.g., Devos et al. 2012; Nosek et al. 2007).

Clinically, the results underscore the need for equitable access to care for gender‐related needs among autistic and non‐autistic gender diverse youth. This is not to suggest that any one form of support, gender‐affirming or otherwise, is most appropriate, or that the different groups require identical approaches to gender‐related care. It may be that support should be tailored for autistic youth to accommodate their autism‐related strengths and difficulties (e.g., Cooper, Mandy, et al. 2023). The point is that the current results suggest clinicians should not assume that gender‐related concerns in autistic youth are necessarily different from those in non‐autistic youth, or amenable to change through support of autism‐specific features alone. Doing so without a more definitive understanding of the pathways to gender diversity in autism would risk misrepresenting gender‐related concerns as secondary to and caused by autism, potentially delaying access to appropriate support.

## Ethics Statement

The study was approved by the Kent Psychology (approval number: 202216553002907588) and HRA and London–Hampstead Research Ethics Committees (reference number: 22/LO/0805).

## Consent

All participants completed the study after providing electronic informed consent or assent.

## Conflicts of Interest

The authors declare no conflicts of interest.

## Supporting information


**Data S1:** Supporting Information.

## Data Availability

The data that support the findings of this study will be openly available on Open Science Framework at https://osf.io/dznkv/?view_only=49159395f86541f5863c937cac1887f2 upon publication.

## Endnotes

^1^
Note that groups differ significantly on verbal IQ. However, when groups were matched for performance on the WASI‐II vocabulary subtest, results did not change substantively (i.e., no significant effect in the original analysis became nonsignificant, and no nonsignificant effect in the original analysis became significant, after groups were matched for verbal IQ).

^2^
In response to a reviewer query, we re‐ran all analyses including sex assigned at birth (male/female) as an additional variable. The results of these analyses, which are presented in the Supporting Information, were entirely consistent with those from the pre‐registered analyses and did not alter conclusions.
