# Supplementary material for: Gender Identity Profiles in Autistic and Non‐Autistic Cisgender and Gender Diverse Youth, and Their Caregivers
Source: Autism Res. 2025 Nov 14;19(1):e70142. doi: 10.1002/aur.70142 (PMC12853228; doi:10.1002/aur.70142)
Supplement: Supplementary file 1 — Data S1: Supporting Information. [file AUR-19-0-s001.docx]

**Gender Identity Profiles in Autistic and Non-Autistic Cisgender and Gender Diverse Youth, and Their Caregivers: Supporting Information**

1. **Matching Procedure for Study 1**

To ensure that group differences reported in the manuscript reflected differences in gender group (gender-referred/cisgender and/or diagnostic status (autistic/nonautistic), rather than differences in sex assigned at birth, age, and FSIQ, we applied a matching procedure using R (version 4.3.3; R Core Team, 2024). Groups were deemed successfully matched if *p*-values were greater than .05 (two-tailed) and effect sizes were small.

Participants were progressively excluded, starting with the youngest individuals from the autistic cisgender, nonautistic cisgender, and nonautistic gender-referred groups, until both the sex ratio and sample sizes were aligned with those of the autistic gender-referred group. In cases where participants within the same group had identical ages, ties were resolved by excluding the participant with the lowest FSIQ from the autistic cisgender and nonautistic gender-referred groups, and the participant with the highest FSIQ from the nonautistic cisgender group. After this step, groups were balanced for sex assigned at birth, age, and FSIQ.

1. **Matching Procedure for Study 2**

In study 2, to match groups for sex assigned at birth and age, the youngest caregivers in each group were progressively excluded, separately for individuals assigned male at birth and those assigned female at birth, until the sample sizes aligned with the group of caregivers of autistic gender-referred youth. Following this, the youngest participants were removed from the cisgender groups, while the oldest participants were excluded from the gender-referred groups. This procedure resulted in groups that were balanced for both sex assigned at birth and age. Note that the matching scripts for both Study 1 and Study 2 were developed by the researchers and are available on the manuscript’s OSF registration page (<https://osf.io/dznkv/?view_only=49159395f86541f5863c937cac1887f2>).

1. **Effect of Sex Assigned at Birth on Key Study Variables**

At the request of an anonymous reviewer, all analyses reported in the main manuscript were reconducted with sex assigned at birth (male/female) included as an additional factor.

In these supplementary analyses on anticipated future gender identity, gender discontentedness, and gender typicality, the only significant effect involving birth-assigned sex was a main effect on gender typicality (birth-assigned females < birth-assigned males; *p* < .001, ƞ_p_^2^ = .06, BF_10_ = 1.14). Importantly, none of the other main or interaction effects involving birth-assigned sex were significant (all *p*s ≥ .070, ƞ_p_^2^ ≤ .02, BF_10_ ≤ 0.48). Thus, autistic and non-autistic participants showed similar patterns of contentedness, anticipated future identity, and typicality, regardless of sex assigned at birth (all sex-involving interactions were small in size, nonsignificant, and associated with Bayes factors that favoured the null). Accordingly, including sex as a factor did not change the conclusions of the primary analyses.

In the analysis of gender dysphoria, the main effect of birth-assigned sex was significant (female > male; *p* = .003, ƞ_p_^2^ = .05, BF_10_ = 0.34), as was the diagnostic status × birth-assigned sex interaction (*p* = .045, ƞ_p_^2^ = .02, BF_10_ = 0.30), although the Bayes factor favoured the null in both cases. No other effects involving birth-assigned sex were significant (all *p*s ≥ .140, ƞ_p_^2^ ≤ .01, BF_10_ ≤ 0.74). Simple main effects analysis of the interaction examined (i) diagnostic status within each birth-assigned sex and (ii) birth-assigned sex within each diagnostic group. These showed that diagnostic status had no significant effect on gender dysphoria in participants assigned female at birth (autistic AFAB: *M* = 2.99, *SD* = 1.60; non-autistic AFAB: *M* = 2.84, *SD* = 1.66), *F*(1, 172) = 1.30, *p* = .257, ƞ_p_^2^ = .01, or male at birth (autistic AMAB: *M* = 2.50, *SD* = 1.46; non-autistic AMAB: *M* = 2.74, *SD* = 1.70), *F*(1, 172) = 2.92, *p* = .089, ƞ_p_^2^ = .02. By contrast, within the autistic group, those assigned female at birth had significantly higher gender dysphoria than those assigned male at birth (autistic AFAB: *M* = 2.99, *SD* = 1.60; autistic AMAB: *M* = 2.50, *SD* = 1.46), *F*(1,172) = 12.86, *p* < .001, ƞ_p_^2^ = .07. However, among non-autistic participants, the difference between participants assigned female at birth and those assigned male at birth was nonsignificant (non-autistic AFAB: *M* = 2.84, *SD* = 1.66; non-autistic AMAB: *M* = 2.74, *SD* = 1.70), *F*(1, 172) = 0.54, *p* = .465, ƞ_p_^2^ < .01.

Even if reliable, these supplementary results are in keeping with those from the pre-registered analyses reported in the main article original and do not alter the central conclusion that autism *per se* does not alter the presentation of gender dysphoria in gender diverse youth. Rather, these supplementary results might add nuance to the main results by suggesting that birth-assigned sex influences gender dysphoria within autistic youth in a way that it does not among non-autistic youth. While it may be informative to further investigate this specific effect on gender dysphoria in future studies, it is important to note that all of these supplementary results be treated with caution. First, they were gained from exploratory analyses and are subject to post hoc interpretations, which risks type I error and biasing the field. Second, and potentially more importantly, the Bayes Factor associated with the sex × diagnostic status interaction was < 0.30, indicating that the data are approximately 3.3 times more consistent with the null hypothesis than with the alternative (i.e., with the hypothesis that sex does *not* influence gender dysphoria uniquely in autistic participants). Thus, we treat this pattern as a tentative signal rather than a confirmed effect, and it does not change our primary conclusions regardless. Preregistered, adequately powered replications are needed to determine whether any birth-assigned-sex–specific modulation of dysphoria in autistic youth is robust.

1. **Robust ANOVAs**

Parametric ANOVAs are robust to non-normality and variance heterogeneity, especially with the equal group sizes in our study (Glass et al., 1972). Nevertheless, at a reviewer’s request, we re-analysed the Study 1 and Study 2 outcomes flagged by diagnostics for non-normality and/or unequal variances by using the WRS2 package in R, which is specifically designed to perform statistical comparisons of heterogeneous and non-normal data by employing trimmed means and heteroscedasticity-consistent estimators (Mair & Wilcox, 2020). Results were essentially identical for every outcome to those from the ANOVAs reported in the main manuscript:

**Study 1 Outcomes Flagged for Non-Normality and/or Heterogeneity of Variances**

**Gender Discontentedness**

A robust 2 (diagnostic status) × 2 (gender group) ANOVA on Question 1 of the Gender Discontentedness measure revealed an effect of gender group, *W* = 548.98, *p* = .001, reflecting higher discontentedness in gender-referred than cisgender children. There was no effect of diagnostic status (autistic = non-autistic; *W* = 0.15, *p* = .699), or an interaction effect (*W* = 0.02, *p* = .898).

The robust analysis of Question 2 of the Gender Discontentedness measure again revealed a significantly stronger desire to be the opposite gender in gender-referred children compared to cisgender children, *W* = 1,060.79, *p* = .001. There was no effect of diagnostic status, *W* = 2.65, *p* = .107, or an interaction effect (*W* = 0.42, *p* = .517)

**Gender Dysphoria**

A robust 2 (diagnostic status) × 2 (gender group) ANOVA for gender dysphoria revealed effects of gender group (gender-referred > cisgender; W = 1,461.78, *p* = .001), diagnostic status (autistic = non-autistic; W = 0.02, *p* = .900) and their interaction (W = 3.97, *p* = .050). Simple main effects analysis of the interaction showed no difference between autistic and non-autistic participants in the cisgender group, *p* = .098, 95% CI: -0.35, 0.03, or the gender-referred group, *p* = .210, 95% CI: -0.11, 0.47. However, gender-referred autistic youth expressed higher gender dysphoria than cisgender autistic youth, *p* < .001, 95% CI: -3.39, -2.81, and likewise gender-referred non-autistic youth expressed higher gender dysphoria than cisgender non-autistic youth, *p* < .001, 95% CI: -3.63, -3.25.

**Study 2 Outcomes Flagged for Non-Normality and/or Heterogeneity of Variances**

**Parent Age**

A robust 2 (diagnostic status) × 2 (gender group) ANOVA for parent age revealed no significant main effects or interaction, all *W*s ≤ 3.45, all *p*s ≥ .07.

**GIDYQ**

A robust 2 (diagnostic status) × 2 (gender group) ANOVA for parent GIDYQ score revealed a significant main effect of gender group, *W* = 6.97, *p* = .01, but no significant main effect of diagnostic status or diagnostic status × gender group interaction, all *W*s ≤ 0.92, all *p*s ≥ .34.

**RCGI**

A robust 2 (diagnostic status) × 2 (gender group) ANOVA for parent RCGI score revealed no significant main effects or interaction, all *W*s ≤ 1.19, all *p*s ≥ .28

Each of these robust ANOVAs replicated the findings reported in the main manuscript and, with *n* = 45 per cell (Study 1) and 51 per group (Study 2), are well powered and maintain excellent Type I error control under non-normality and variance heterogeneity. Accordingly, these analyses provide a robust complement to the parametric ANOVAs and reinforce the stability of our conclusions (see Erceg-Hurn & Mirosevich, 2008; Keselman et al., 2008; Mair and Wilcox, 2020).

**References**

Erceg-Hurn, D. M., & Mirosevich, V. M. (2008). Modern robust statistical methods: An easy way to maximize the accuracy and power of your research. American Psychologist, 63(7), 591–601. [https://doi.org/10.1037/0003-066X.63.7.591](https://psycnet.apa.org/doi/10.1037/0003-066X.63.7.591)

Glass, G. V., Peckham, P. D., & Sanders, J. R. (1972). Consequences of failure to meet assumptions underlying the fixed effects analyses of variance and covariance. Review of Educational Research, 42(3), 237–288. <https://doi.org/10.3102/00346543042003237>

Keselman, H. J., Algina, J., Lix, L. M., Wilcox, R. R., & Deering, K. N. (2008). A generally robust approach for testing hypotheses and setting confidence intervals for effect sizes. Psychological Methods, 13(2), 110–129. [https://doi.org/10.1037/1082-989X.13.2.110](https://psycnet.apa.org/doi/10.1037/1082-989X.13.2.110)

Mair, P., & Wilcox, R. (2020). Robust statistical methods in R using the WRS2 package. Behavior Research Methods, 52(2), 464–488. <https://doi.org/10.3758/s13428-019-01246-w>

| **Table S1**  *Study 1: Deviations from Pre-Registration* | | |
| --- | --- | --- |
| **Where?** | **What?** | **Why?** |
| Sample size | - Final sample: 45 participants in each group, rather than 50 per group. | - High attrition rate, the closure of the UK’s only national gender identity service, and general recruitment challenges. |
| Data collection | - Age range 7–16 years instead of 7–14 years - Use of social media to recruit both cisgender and gender-referred participants. | - To maximise the chances of achieving the target sample size, and consequently, ensure adequate statistical power to conduct the planned statistical analyses. |
| Data exclusion | - Gender-referred participants undergoing an ASD assessment were included in the autistic gender-referred group. - Gender-referred children who reported a gender identity congruent with their sex assigned at birth were included in the gender-referred group. | - Due to difficulties meeting the target sample size for the gender-referred groups. |
| Data analysis | - Data on future gender identity were analysed via a binary logistic regression. | - A planned analysis was not specified for this analysis due to an oversight in the pre-registration. |

| **Table S2**  *Study 2: Deviations from Pre-Registration* | | |
| --- | --- | --- |
| **Where?** | **What?** | **Why?** |
| Sample size | Final sample: 50 autistic gender-referred, 51 autistic cisgender, 51 nonautistic gender-referred, and 51 nonautistic cisgender, rather than 50 per group. | - To maximise statistical power, additional caregivers, initially recruited for matching purposes, were included in the sample. |
| Data analysis | Between-group differences in sex ratio were examined conducting a series of Fisher’s exact tests, rather than a 3-way log-linear analysis. | - Due to the small number of caregivers assigned male at birth in the sample, some expected cell counts were below 5, so the planned analysis could not be conducted. |
